# The pot calling the kettle black: the extent and type of errors in a computerized immunization registry and by parent report

**DOI:** 10.1186/1471-2431-14-1

**Published:** 2014-01-04

**Authors:** Shannon E MacDonald, Donald P Schopflocher, Richard P Golonka

**Affiliations:** 1 Department of Pediatrics, Faculty of Medicine, University of Calgary, 2888 Shaganappi Trail NW, Calgary, Alberta T3B 6A8, Canada; 2 Faculty of Nursing, University of Alberta, Level 3, Edmonton Clinic Health Academy, 11405-87 Ave, Edmonton, Alberta T6G 1C9, Canada; 3 School of Public Health, University of Alberta, 3–300 Edmonton Clinic Health Academy, 11405- 87 Ave, Edmonton, Alberta T6G 1C9, Canada; 4 Alberta Health Services, Health Protection, Communicable Disease Control, Main Floor, West Tower Coronation Plaza, 14310-111 Ave, Edmonton, Alberta T5M 3Z7, Canada

**Keywords:** Immunization, Vaccination, Immunization status, Immunization information system (IIS), Registry, Parent report, Misclassification

## Abstract

**Background:**

Accurate classification of children’s immunization status is essential for clinical care, administration and evaluation of immunization programs, and vaccine program research. Computerized immunization registries have been proposed as a valuable alternative to provider paper records or parent report, but there is a need to better understand the challenges associated with their use. This study assessed the accuracy of immunization status classification in an immunization registry as compared to parent report and determined the number and type of errors occurring in both sources.

**Methods:**

This study was a sub-analysis of a larger study which compared the characteristics of children whose immunizations were up to date (UTD) at two years as compared to those not UTD. Children’s immunization status was initially determined from a population-based immunization registry, and then compared to parent report of immunization status, as reported in a postal survey. Discrepancies between the two sources were adjudicated by review of immunization providers’ hard-copy clinic records. Descriptive analyses included calculating proportions and confidence intervals for errors in classification and reporting of the type and frequency of errors.

**Results:**

Among the 461 survey respondents, there were 60 discrepancies in immunization status. The majority of errors were due to parent report (n = 44), but the registry was not without fault (n = 16). Parents tended to erroneously report their child as UTD, whereas the registry was more likely to wrongly classify children as not UTD. Reasons for registry errors included failure to account for varicella disease history, variable number of doses required due to age at series initiation, and doses administered out of the region.

**Conclusions:**

These results confirm that parent report is often flawed, but also identify that registries are prone to misclassification of immunization status. Immunization program administrators and researchers need to institute measures to identify and reduce misclassification, in order for registries to play an effective role in the control of vaccine-preventable disease.

## Background

In order to optimize the effectiveness and safety of childhood immunization programs it is essential to have an accurate record of children’s immunization status, and ultimately population-level immunization coverage. Such information is essential for clinical care, administration and evaluation of immunization programs and policies, vaccine research, and tracking vaccine-associated adverse events [1-7].

Accurate assessment of immunization status is dependent on having a valid, comprehensive, and accessible source of data [8]. While hard-copy provider records (typically clinic charts) are a trusted source of immunization status [9,10], they are not a feasible, cost-effective, or easily accessible method for tracking individual or population level coverage on an ongoing basis [11]. Parent-held records or parent recall are alternatives that are commonly used in immunization research, but these sources are often inaccurate; parent-held records typically underestimate coverage, while parent recall tends to overestimate it [8,9,12-15]. Population-based electronic immunization registries, also known as Immunization Information Systems (IIS), have been proposed as a valid, cost-effective, and accessible option for assessing immunization status [1,6,16]. These registries are centralized electronic repositories for immunization data for a specified geographic location that can consolidate immunization records from multiple providers and settings [1]. They have been promoted by immunization advisory bodies in the USA and Canada [17-20], and have been proposed as an alternative source of immunization status verification for the National Immunization Survey conducted annually in the USA [21]. However, it has been recognized that additional validation studies are needed to determine the accuracy of registry data and identify areas for improvement [1,16,21,22].

The purpose of this article is to: (a) report on the accuracy of immunization status classification in an immunization registry as compared to parent report, and (b) identify the frequency and type of errors for both sources, in order to identify areas for system improvement.

## Methods

### Study design

This study was a sub-analysis of a larger research study conducted in 2009–2010 investigating various factors associated with childhood immunization uptake. The study utilized a postal survey to assess the immunization knowledge and experiences of parents of children whose immunizations were up to date (UTD) at age two, as compared to those who were not UTD. The approval of the Health Research Ethics Board at the University of Alberta and participant informed consent were obtained for the study. The details of the postal survey and the results of the multivariate model of factors influencing uptake are currently under review for publication elsewhere.

### Study setting and population

The study population for the postal survey was selected from a regional immunization registry in the city and surrounding rural communities of Edmonton, Alberta (population 1.1 million) in Canada. This registry includes immunization data on all children who were born in the Edmonton zone, as well as those that moved to the zone and accessed public health services. All routine childhood immunizations in this zone are administered by nurses in community-based public health clinics, recorded on a hard-copy clinic record, and entered in the electronic registry onsite by designated clerical staff. Each child is assigned to a ‘home’ public health clinic where their chart is stored. If an appointment is made at a different location, the chart is transferred prior to the visit, any immunizations administered are recorded on the chart and entered into the registry on site, and then the chart is transferred back to the home clinic. The registry software only permits entry of a vaccine dose with a ‘valid date of administration' , i.e. a specific date must be attached to each vaccine dose for it to be entered in the registry.

To be considered UTD, children had to have received all doses of the five vaccines recommended in the Alberta provincial schedule at that time: Diphtheria, Tetanus, acellular Pertussis, Polio and Haemophilus influenzae type b (4 doses); Measles, Mumps, Rubella (1 dose); Varicella (1 dose or history of disease); Meningococcal C conjugate (3 doses); and Pneumococcal 7-valent conjugate (4 doses). This schedule is not consistent across Canada since each province and territory sets its own immunization schedule. The study accounted for variation in the number of doses required for children who were older at initiation of the series or due to individual clinical conditions (e.g. if first dose of meningococcal vaccine is given between 4 and 12 months old, only two doses are required).

### Sampling

Children were randomly selected from a one year birth cohort (born May 1, 2006 – April 30, 2007) in the regional immunization registry. An algorithm was used to identify children who were UTD (n = 371) or not UTD (n = 371) at two years of age. The sample size was determined using an effect size and response rate from a previous study [23], a 95% Confidence Interval, 80% Power (ß = 0.20), and a 1:1 ratio of cases to controls.

### Postal survey administration

Parents of children identified from the registry were mailed a postcard informing them about the study, followed by the survey a week later. If no response was received after 3 weeks, a reminder postcard was sent, followed by a replacement survey 3 weeks later. In addition to asking about their beliefs and experiences with immunizations, the survey asked parents to report their child’s immunization status according to parent-held records or recall. Specifically, parents were asked whether their child had: (a) not received any immunizations; (b) received some, but not all, the immunizations for their age; or (c) received all the immunizations for their age. Parents were not required to consult the parent-held record due to concerns that excessive participant burden might adversely impact the response rate.

### Confirmation of immunization status and data analysis

In cases where there was a discrepancy in immunization status between the registry and parent report, the original hard-copy clinic record was reviewed. The clinic chart was considered the ‘Gold standard’. Given that immunizations are documented on this clinic chart at time of administration, errors in this record are unlikely and no other data source would have superior accuracy. When the parent report of immunization status agreed with that in the registry, no confirmation using clinic charts was undertaken. For this sub-analysis of the larger study, proportions and confidence intervals for errors in reporting were calculated and the type and frequency of errors were determined.

## Results

### Survey response

Of 1342 potential study subjects, 274 were ineligible due to invalid addresses, 18 withdrew from the study, 589 did not respond, and 461 (331 UTD and 130 not UTD) returned a completed questionnaire. After removing undeliverable surveys from the denominator, the final response rate was 43% (461/1068). Respondents were more likely to be UTD (71.8% UTD, 95% CI: 67.5% - 75.7%, 331/461) as compared to of non-respondents (50.9% UTD, 95% CI: 46.9% - 55.0%, 300/589).

### Amount and reasons for error

There were a total of 60 discrepancies between the registry and parent report of immunization status among the 461 survey respondents. Chart review indicated that 44 children were misclassified due to parent reporting and 16 due to registry errors. Table 1 presents the number and direction of misclassification errors from each source. None of the 315 children identified as UTD by the registry were misclassified, while 11.0% (95% CI: 5.9% - 16.0%) of the children recorded as not UTD by the registry (16/146) were misclassified, i.e. they were reported as UTD by parents and confirmed by chart review. The level of error for parent report was the inverse, 11.3% (95% CI: 8.1% - 14.5%) of children reported as UTD by parent report (42/371) were considered not UTD by the registry and confirmed by clinic chart review, while only (2.2%, 95% CI: 0.8% - 5.3%) of the children reported as not UTD by parents were actually UTD (2/90). The frequencies of specific reasons for misclassification from each data source are provided in Table 2.

**Table 1:** Registry and parent report versus clinic record

		**Clinic record (Gold standard)**		
		**UTD**	**Not UTD**	**Total**
**Registry**	**UTD**	315^a^	0^a^	315
	**Not UTD**	16^b,c^	130^a^	146
	**Total**	331	130	461
**Parent report**	**UTD**	329^a^	42^b,c^	371
	**Not UTD**	2^b,c^	88^a^	90
	**Total**	331	130	461

^a^Registry and parent report agreed, so no clinic record review conducted.

^b^Confirmed by clinic record review.

^c^Indicates misclassification errors.

**Table 2:** Number and types of misclassification errors

			
**Types of errors in registry**			**n**
	Dose not entered in registry		1
	History of varicella disease not entered in registry		4
	Fewer doses required due to age at first dose, but not noted in registry		2
	Doses given out of region, but not entered in registry		5
	Child moved from another province and considered complete by other province’s schedule, but not noted in registry		4
**Subtotal**			16
**Types of errors in parent reporting**			**n**
	Parent reported child UTD, although:		
		Missed dose(s) (unexplained intentional or unintentional misreporting)	25
		Refused varicella vaccine	6
		Did not complete immunizations until after received survey	11
	Parent reported child not UTD, although actually complete (unexplained intentional or unintentional misreporting)		2
**Subtotal**			44
**Total number of errors from all sources**			60

## Discussion

### Differential accuracy of reporting and its implications

The results of this study confirm previous findings in other settings that parent reporting of immunization status is not always accurate [8,9,12-15], but also identifies potential limitations of immunization registry data. The amount of error in the registry found in this study was 3.5%; 16 of 461 immunization records in the final study sample (presuming there were no errors in concordant records that were not checked). This is likely somewhat reassuring to the registry administrators, who consider <3% to be an acceptable amount of error. However, if registries are to be heralded as the most accurate and reliable source for tracking immunization coverage in the future, this level of error is noteworthy when interpreting coverage calculations.

The differential accuracy of reporting children UTD versus not UTD by parent report and the registry is of particular interest (see Table 1). Specifically, we found that parents were generally very accurate in reporting their child as not UTD (only 2.2% were misclassified), but not for reporting them as UTD (11.3% were misclassified). In contrast, the registry was extremely accurate when recording a child as UTD (no errors), but less so when not UTD (11% were misclassified). Only two previous studies have simultaneously compared registry and parent reporting to a third Gold standard [10,24], and only one [10] described the differential accuracy of UTD/not UTD for the registry versus parent report.

These findings have important implications for program administration, clinical follow-up of individual children, and vaccine research. When evaluating immunization program effectiveness, any child classified as not UTD in a registry may need to be verified by chart review before drawing conclusions about coverage levels for a given region or clinic. In terms of clinical care, a child classified as not UTD in the registry should be verified in the clinic chart before recall/reminder notices are sent to parents. Further validation of immunization through consultation with parents before additional doses of vaccine are administered would avoid administering “extra” vaccine doses, as well as contribute to a self-correcting process to improve registry accuracy. In our study, no children were actually “over-immunized” due to registry error because registry information is always confirmed in the clinic chart for pre-school aged children before vaccines are administered. However, errors could occur after the child turns 5 years, as the chart is then moved into storage and immunization providers utilize the registry for clinical care decisions. In other settings with similar protocols, or where multiple immunization providers access the registry in a clinical setting (i.e. at point of care), it is entirely possible that children could be “over-immunized” based on erroneous registry information. In contrast, when a parent presents their child for medical care and reports their child as *not* UTD for immunizations, they can be considered generally accurate and appropriate follow-up, including supplementary doses, should be pursued. For research purposes, such as vaccine effectiveness or adverse event studies, our findings suggest that a registry may be the best option for sample selection if the aim is to include both UTD and not UTD children in a study, given the lower overall error rate, as compared to parent report. A registry is also preferable if only UTD children are being studied (no misclassification, compared to parent report), whereas parent report would be a more valid source for identifying subjects if the primary focus is not UTD children.

### Reasons for misclassification

Perhaps the most valuable contribution of this study is new knowledge about the specific types of errors contributing to misclassification in immunization registries and parent report (see Table 2). Few studies have reported this information, yet this is a critical element in identifying potential improvements to parent/public health education, quality control, follow-up of incompletely immunized children, and system improvements.

The types of registry errors identified in the limited number of previous studies include: errors in transcription of number or dates of doses administered, and errors in transcribing vaccine formulation, manufacturer, or lot number [25,26]. Our study is the first to identify *failure to transcribe varicella disease history* as a considerable source of registry error (accounting for 25% of registry errors). *Failure to transcribe factors that reduce the required number of vaccine doses*, including late initiation of vaccine series and doses given out of the region, were also a significant source of error (> 40% of registry errors). Registry errors due to children moving from another province and being considered complete by the other province’s schedule (another 25% or registry errors) is a problem unique to the Canadian context, where immunization schedules can vary substantially between jurisdictions. It is noteworthy that the registry used in our study was probably more comprehensive (i.e. fully inclusive of the target population) than in other settings, due to the one-provider system for immunizations in Alberta; thus errors identified in our study might be further compounded in multi-provider settings due to record-scattering [27,28].

Identifying the reasons for errors in registry data is crucial in determining strategies for improvement. Earlier studies have found that non-transcribed data are sometimes found in locations not routinely transcribed (e.g. discharge summaries, encounter notes) or are in a format not conducive to transcription (e.g. stated as ‘up-to-date' , but no specific dates given) [29]. Our study found similar issues, as well as possibly a lack of awareness by data entry clerks that notations, such as varicella disease history and age at first dose, are relevant data in determining immunization status. These problems with transcription of charted data identified in ours and other studies [25,26,29], suggest the need for adequate training of data entry personnel and the need to assess the format of charting forms to facilitate consistent recording of relevant data in the appropriate location for ease and completeness of transcription. Administrators of immunization registries can aid in assuring and improving data accuracy by adopting strategies to decrease the potential for misclassification, including direct electronic data entry [30,31], electronic data transfer [10,25], double data entry [26], and audit procedures [16].

The types of errors in parent reporting of immunization status are also of interest. Although the majority of errors (more than 50%) were unexplained, and are likely due to intentional or unintentional misreporting, other types of errors suggest the need for parent education and system improvements. The fact that some parents (14%) who refused varicella vaccine still considered their child to be UTD suggests that these parents did not recognize this vaccine as part of the ‘routine’ immunization series. This is congruent with findings in our larger study, that many parents are selectively refusing the varicella vaccine because they do not consider it an ‘essential’ element of routine childhood immunizations. Another interesting finding was that a number of parents (25% of parent errors) who had not completed their children’s immunizations (more than 6 months after the final dose was due) did so after receiving the survey in the mail. Presumably the survey acted as a reminder mechanism for completion of the series, which speaks to the need for effective follow-up of non-UTD children in this setting and the value of reminder/recall systems.

### Strengths and limitations

This study has a number of specific strengths that enable it to contribute new and valuable knowledge regarding the accuracy of immunization registries. This is one of the few studies comparing two alternate sources of data on immunization status to a third Gold standard and is the only published Canadian study to assess the accuracy of an immunization registry. The one-provider system for immunizations in this setting was a particular strength as it virtually eliminated the possibility that ‘record-scattering’ of provider records biased the Gold standard [27,28]. While the uniqueness of this system might influence the magnitude of data errors (i.e. underestimating what may occur in multi-provider systems), the implications of such reporting errors and the reasons for misclassification that we identified have broad applicability to other regions utilizing immunization registries for surveillance and clinical care.

There were some limitations to this study, which need to be considered in the interpretation of the findings. As study subjects were selected on the basis of immunization status, we were unable to make inferences about prevalence of UTD/not UTD status, and since subjects were not selected on the basis of the Gold standard, reporting of sensitivity, specificity, and predictive values would be misleading. We accept this limitation since a case–control study was the best design to obtain a substantial number of not UTD children in a population with relatively highly immunization coverage [32], and because there is a recognized need to assess data accuracy in immunization program research of all study designs, not merely cohort and cross-sectional studies [1]. The fact that registry accuracy was only assessed for respondents to the survey suggests the potential for bias in this study. Typically survey non-respondents are more likely to be not UTD [10], as was true in our study. Since registry errors were more common for not UTD records, our study likely under-estimates the number of registry errors. Finally, an assumption was made that only incongruent reports of immunization status between parent and registry data need be adjudicated by clinic charts. This assumption is considered justified, since the likelihood of registry and parent errors being in the ‘same direction’ (i.e. both erroneously UTD or not UTD) is minimized due to the fact that (a) the registry software only accepts doses with a specified date, which largely limits mistakes to ‘errors of omission’ [26], and (b) parents who report their child as not UTD are typically accurate [11,33]. Nonetheless, we recognize that without adjudicating all 461 records, this assumption does leave a source of potential bias.

## Conclusions

Despite the significant benefits of population-based immunization registries, our study highlights the potential challenges in ensuring the accuracy of this data source. Clearly, registry records should not always be presumed superior to parent report. At the individual level, parents may be more accurate at identifying their child as not UTD, while the registry is more accurate at identifying UTD children. At the population level, coverage derived from the registry may *under*-estimate coverage rate, while parent reports tend to *over*-estimate coverage.

Studies such as this one contribute knowledge needed to improve the quality, completeness, and regional comparability of immunization registries before they can be considered a valid and reliable source of data on immunizations status [21]. We strongly recommend further targeted studies of registry data accuracy in other settings. We also suggest that researchers utilizing immunization registries conduct a quality assessment of their data source, including using appropriate algorithms to confirm classification of immunization status and/or assessing a random sample of subjects in the registry to ascertain the accuracy of the registry versus a Gold standard.

The ultimate goal of any immunization tracking system is to improve the protection of the population from vaccine-preventable disease. As childhood immunization schedules become more complex, parent reporting is likely to become less and less accurate; and as provider records become more and more scattered due to our increasingly mobile society [34], registries have the potential to be not only the best, but the only viable method for tracking individual and population level coverage. This increasing dependence on registries can lead to improvements in population and individual health if appropriate measures are instituted to ensure the accuracy of this data source.

## Competing interests

The authors declare that they have no competing interests.

## Authors’ contributions

SM conceived and designed the study, collected the postal survey data, analysed and interpreted the data, and drafted and revised the manuscript. DS was involved in the study design, data analysis, and the outline and revision of the manuscript. RG was involved in the sample selection, interpretation of the data, and made substantive contributions and revisions to the manuscript. All authors read and approved the final manuscript.

## Pre-publication history

The pre-publication history for this paper can be accessed here:

http://www.biomedcentral.com/1471-2431/14/1/prepub
